# Neural basis of cognitive control signals in anterior cingulate cortex during delay discounting

**DOI:** 10.1101/2024.06.07.597894

**Published:** 2024-06-08

**Authors:** Jeremy K. Seamans, Shelby White, Mitchell Morningstar, Eldon Emberly, David Linsenbardt, Baofeng Ma, Cristine L. Czachowski, Christopher C. Lapish

**Affiliations:** 1 Stark Neuroscience Institute, Department of Anatomy, Cell Biology, and Physiology, Indianapolis, 46202, USA; 2 Indiana University-Purdue University, Indianapolis, Psychology Department, Indianapolis, 46202, USA; 3 University of New Mexico, Department of Neurosciences, Albuquerque, 87131, USA; 4 Department of Physics, Simon Fraser University, Burnaby, BC, V5A 1S6; 5 Dept of Psychiatry, Djavad Mowafaghian Centre for Brain Health, 2211 Wesbrook Mall, UBC, Vancouver BC, V6T2B5

## Abstract

Cognitive control involves allocating cognitive effort according to internal needs and task demands and the Anterior Cingulate Cortex (ACC) is hypothesized to play a central role in this process. We investigated the neural basis of cognitive control in the ACC of rats performing an adjusting-amount delay discounting task. Decision-making in this this task can be guided by using either a lever-value tracking strategy, requiring a ‘resource-based’ form of cognitive effort or a lever-biased strategy requiring a ‘resistance-based’ form of cognitive effort. We found that ACC ensembles always tightly tracked lever value on each trial, indicative of a resource-based control signal. These signals were prevalent in the neural recordings and were influenced by the delay. A shorter delay was associated with devaluing of the immediate option and a longer delay was associated with overvaluing of the immediate option. In addition, ACC theta (6–12Hz) oscillations were observed at the choice point of rats exhibiting a resistance-based strategy. These data provide candidates of neural activity patterns in the ACC that underlie the use of ‘resource-based’ and ‘resistance-based’ cognitive effort. Furthermore, these data illustrate how strategies can be engaged under different conditions in individual subjects.

## Introduction

Cognitive effort is commonly thought of as being equivalent to mental exertion, however multiple types of cognitive effort have been identified and formalized^1,2^. A *resourced-based* form of cognitive effort is relevant whenever a task relies on a valuable but depleting resource, such as attention or working memory^1,3,4^. There is also a *resistance-based* form of cognitive effort that is used to overcome some type of internal ‘resistive force’, such as unpleasantness or impatience^2,5^. Both types of cognitive effort are costly and are typically avoided when possible. However, the perceived costs of deploying cognitive effort must be weighed against the consequences of not exerting effort. Such decisions are influenced by a variety of extrinsic and subjective factors, including the task demands, the individual’s propensity for one type of cognitive effort over another, their level of arousal or fatigue, and the urgency of the need that would be satisfied by exerting the effort^2,5^. For these reasons, cognitive effort cannot be measured in purely objective terms but must be inferred from a subject’s behavior or from physiological measures.

Decision-making tasks involving intertemporal choices have commonly been used to study cognitive effort in both humans^4^ and rats^6,7^. These tasks require subjects to choose between accepting a low-value reward immediately or waiting for a higher valued reward to be delivered after a delay. The adjusting amount delay-discounting task^8,9^ adds an additional layer of complexity, as the payout for the immediate option decreases whenever the immediate option is chosen and increases whenever the delayed option is chosen. In order to both maximize reward and minimize waiting, the subject should generally favor the delayed lever option but exploit the immediate lever option whenever its value (i.e. payout) is high. As a result, these tasks require a combination of the two types of cognitive effort mentioned above: The resistance-based form is needed in order to overcome the unpleasantness of waiting out the delays associated with the delayed lever option, whereas the resourced-based form cognitive effort is necessary when the subject must draw on attentional and mnemonic resources to keep track of the value of the immediate lever option to know when it’s value is high.

Deciding how and when to deploy cognitive effort is a form of cognitive control that is thought to rely primarily on the ACC. Cognitive control processes, mediated by the ACC, dynamically regulate how much and what type of cognitive effort should be deployed by comparing the expected value of the outcome produced by the effort versus the cost of implementing and maintaining that effort^1,10^. In this regard, the cost of cognitive effort is ‘felt’ at a physiological level through changes in emotion and autonomic tone^2,4,11–13.^

The ACC is well suited to track the cost of cognitive effort due to its extensive bilateral connections with regions involved in regulating autonomic tone^14–16^. There is also considerable support for the idea that the ACC is involved in effort-based decision-making more generally as multiple studies have shown that ACC activity represents the degree of expected effort^17–19^ and that ACC lesions in rodents reduce the propensity to make choices that involve physical effort, even if such efforts result in higher rewards^20–22^. In line with this view, inactivation of the ACC reduces the willingness of rats to expend cognitive effort to obtain larger rewards on a variant of the 5-choice serial reaction time task^23^. In that study, cognitive effort was defined based on the relative demands placed on visuospatial attention, which is an example resourced-based cognitive effort.

The neural mechanisms of cognitive control have been studied at a macroscopic level in humans using EEG^24,25^ and theta oscillations have emerged as a candidate signal. Frontomedial theta oscillations originate in the ACC and potentially synchronize ACC with other brain regions when control is deployed^26,27^. Fronotmedial theta exhibits several features that would be expected of a cognitive control signal, as it increases following negative feedback, over time in tasks that require sustained effort, and under conditions when a decision is difficult^28,29^. However, the cellular mechanisms within the ACC that deploy and control various types of cognitive effort have not been identified.

We sought to address this knowledge gap by recording from ensembles of ACC neurons during the adjusting amount delay-discounting task. The task is well suited for this purpose since it involves the two forms of cognitive effort described above. If rats opt to make choices based on the relative values of the two levers, they would need to rely on a resource-based form of cognitive effort in order to keep track of the ever-changing value of the immediate lever (ival). This strategy would be expressed as a switch from focusing on delayed lever-presses (dLPs) when ival was low to immediate lever-presses (iLPs) when ival was high. Alternatively, a rat may decide to focus solely on dLPs throughout the session. While this strategy forgoes the need for ival tracking, it does require a strong reliance on a resistance-based form of cognitive effort in order to endure the delays associated with each dLP. Neural and behavioral correlates of each strategy was assessed in the DD task.

## Methods

### Subjects and task

For electrophysiology experiments, 10 male Wistar rats were purchased from Envigo (Indianapolis, IN). Animals were acclimated to vivarium conditions, a 12-h reverse light/dark cycle with lights ON at 7:00 PM, for 3 days prior to handling. Animals were then single-housed with ad lib access to food and water for a week and were at least 70 days of age prior to food restriction and habituation to the task. All procedures were approved by the IUPUI School of Science Institutional Animal Care and Use Committee and were in accordance with the National Institutes of Health Guidelines for the Care and Use of Laboratory Animals.

### Apparatus

Behavioral training was performed in eight standard one-compartment operant boxes (20.3 cm × 15.9 cm × 21.3 cm; Med Associates, St Albans, VT) inside of sound attenuating chambers (ENV-018M; MED Associates, St. Albans, VT). Each box contained one wall with two stimulus lights, two retractable levers that flanked a pellet hopper, and a tone generator. Cue lights were above each lever. A tone generator (2900 Hz) was above the hopper. A house light was on the opposite wall.

All awake-behaving electrophysiological recordings were performed in one custom-built operant box (21.6 cm × 25.7 cm × 52.0 cm). Dimensions, stimuli (including house and cue lights), and retractable levers were all positioned to replicate the conditions of the operant boxes as closely as possible. The floor bars of the custom-built box were made of painted wooden dowels. All metal components of the box were powder coated. MED-PC IV software (Med Associates, St. Albans, VT) was used to all environmental variables (e.g. lever extensions, presses, lights on/off).

### Behavioral Training

Following a week of single housing, animals were handled daily for a week. Animals were food restricted to 85% of their starting free-feeding weight and maintained under this condition for the duration of the experiment except for immediately before and after surgery. Animals received their daily amount of food following testing.

Animals were habituated on Day 1 to the operant chambers for 30 minutes. Shaping procedures began on Day 2 and details for shaping procedures can be found in^30^. In brief, animals were trained to press each lever for a sucrose pellet and environmental variables were introduced in a staged manner over 6 days. Learning the contingency between pressing the lever and receiving a pellet, was demonstrated by a minimum of 30 successful reinforced lever responses for each lever, which was typically completed within 6 days.

On days 7 and 8 training on the task with all environmental stimuli was initiated. Illumination of the house light signaled the start of a trial and remained on for 10 seconds. Once extinguished, both levers extended, and the animal was required to press either lever in order to initiate the start of the trial. No response for 10 seconds resulted in retraction of the levers followed by the illumination of the house light (10 seconds). Once a lever was pressed to initiate the trial, both levers retracted for 1 second. Levers were reinserted into the chamber and lights above each lever were illuminated. A response on either lever was marked with a 100ms tone and a single sucrose pellet delivered, simultaneously. Only the cue light above the chosen lever remained on for the remainder of the trial. The duration of the trials was always 35 seconds. These sessions were terminated either when 30 choices were made or when 35 minutes had elapsed. Over sessions 7 and 8, lever preference bias was determined for each animal.

### Delay Discounting Task

The within-session adjusting amount DD procedure was a modified version of the procedure^30^ which was originally adapted from Oberlin and Grahame^31^. Stimuli were presented in the exact manner detailed in days 7 and 8 of shaping except that the number of pellets delivered for a given trial was dependent on lever pressing contingencies detailed below. The “delay lever” was assigned to each animal as their non-preferred side. Choosing the delay lever always resulted in the delivery of 6 pellets following some delay (0, 1, 2, 4, or 8s). Choosing the immediate lever resulted in 0–6 pellets delivered immediately (i.e. the adjusting amount lever). The value of the immediate lever (ival), was defined as the number of pellets delivered by the immediate lever. Ival was always set to 3 pellets at the start of each session. On “choice trials” each response on the immediate lever would decrease the number of pellets the immediate lever would dispense on the next trial by one (minimum 0 pellets) whereas a response on the delay lever would increase the number of pellets the immediate lever would dispense on the next trial by one (max 6 pellets). “Forced trials” were implemented for the immediate and delay levers, where two consecutive responses on the same lever would result in a forced trial for the non-chosen lever on the next trial. If an animal did not lever press on the forced trial, the forced trial would be presented again until the lever was pressed. The animal had to eventually make a response on the forced trial in order to return to choice trials. There was no effect of forced trials on the value of the immediate lever.

Animals then completed 0, 1, and 2-sec delays in ascending before surgery. After completing a delay, animals had one day off where no testing occurred. Eight to twelve sessions were given at the 0-sec delay and four sessions at the 1 and 2-sec delay. Reward magnitude discrimination was determined at the 0-sec delay in the standard operant chambers using the 45mg sucrose pellets with an exclusion criterion of 70% (4.2 pellets) of the maximum reward value (6 pellets). Magnitude criteria were meant to assess whether animals understood the lever contingencies before moving forward with subsequent delays, specifically, that there was no penalty for pressing the delay lever at a 0-sec delay.

Once the animals recovered from surgery (see below) they were given a 2-sec delay ‘reminder session’ before recording neural activity. Each recording session consisted of 40 choice trials or 45mins and used 20mg sucrose pellets.

### Electrophysiology Surgical Preparation & Implantation

Animals were anaesthetized with isoflurane gas (2% at 4L/h) until sedated, at which point they were placed in a stereotaxic frame and maintained on 0.3–0.5% isoflurane for the duration of the surgery. Artificial tears were then applied. Subsequently, fur was shaved and the skin at the incision site was sanitized with three rounds of both 70% EtOH and betadine before applying a local anesthetic (Marcaine; 5mg/kg s.c.). An anti-inflammatory (Ketofen; 5mg/kg dose s.c.) and antibiotic (Cefazolin; 30mg/kg s.c.) were injected at the nape of the neck (anti-inflammatory and antibiotic) before beginning the incision. Once the skull was exposed and cleaned of blood, bregma-lambda coordinates were identified. Prior to implantation of Cambridge Probes, four anchoring screws were inserted.

A small, rectangular craniotomy was performed over the right hemisphere of MFC (AP: 2.8, ML: 0.3 from bregma) followed by a durotomy and cleaning/hydration of the probe insertion site with a sterile saline solution. Additionally, two ground screws were placed above the cerebellum. A Cambridge Neurotech F (n=5), P (n=4), or E-series (n=1) 64-channel silicon probe on a movable drive (Cambridge Neurotech, Cambridge, UK) was lowered to the target site. Mobility of the movable drive was maintained with a coating of antibiotic ointment. Following insertion of Cambridge Probes, a two-compound dental cement was used to adhere implants to anchoring screws. Following completion of surgical procedures, animals were maintained in a clean heated cage and monitored for recovery before being returned to the vivarium.

### Electrophysiology Equipment

An electrical interface board (EIB) connected silicon electrodes to an Intan Omnetics headstage (Intan – CA). An Intan RHD SPI cable (Intan – CA) connected the headstage to a Doric Commutator (Doric Lenses – Canada) positioned above the operant apparatus. An OpenEphys (OpenEphys – MA) acquisition system was used to collect all electrophysiological data. Data was streamed from the OpenEphys acquisition box to a compatible desktop computer via a USB 2.0 connection and sampled at 30 kHz. AnyMaze (ANY-maze Behavioral tracking software – UK) was used to collect all behavioral and locomotor data. ANY-maze locomotor data was synchronized with OpenEphys via an ANY-maze AMI connected to an OpenEphys ADC I/O board. Med PC behavioral events were also synchronized to the electrophysiological recordings via an OpenEphys ADC I/O board. Following sessions with diminished signal, electrodes were lowered 50μm following completion of that session in order to allow any drifting of the probe to occur before the next day’s session.

### Analyses of Electrophysiology Animals

The goal was to group the sessions based on whether a ‘dLP biased’ strategy, ‘ival-tracking’ strategy, or no dominant strategy was employed. Choices were separated based on whether the rat pressed the lever that delivered a variable number of pellets immediately (immediate lever presses, iLPs) or 6 pellets after a delay of 4 or 8s (delayed lever presses, dLPs). The relative proportion of dLPs versus iLPs on free trials was then calculated for trials in which ival was low (0–2), medium (3–4) or high (5–6). This produced a 3 × 54 matrix that was submitted to k-means (with k=3) for clustering. K-means effectively separated the sessions based on behavioral strategy, with G1 sessions exhibiting a strong dLP biased strategy (i.e. a high dLP:iLP at all ivals), G2 sessions exhibiting an ival-tracking strategy (i.e. a high dLP:iLP when ival was<3 but a low dLP:iLP when ival was >4), and G3 sessions which exhibited a mixture of the 2 strategies. For interpretability of the results, it was also important ensure that all sessions within a group had a consistent delay interval. In order to ensure this was the case while not biasing clustering with an explicit delay term, 3 G1 sessions with an 8s delay and 2 G2 sessions with a 4s delay were excluded from further analyses. The breakdown of sessions per rats in the 3 session groups is shown in Table 1. Note that most animals moved between the groups and therefore the inferences made on impulsivity reflect state rather than trait variables.

### Reinforcement Learning (RL) Framework: Behavioral analysis and simulation of behavior

The task was captured by a model with 7 states and 12 actions (see methods, Figure 2A). Here an ‘action’ was a LP and a ‘state’ could be conceived of as any time between pairs of LPs. Although the task state map could be arranged in a number of possible configurations, 7 states were chosen because this was the minimal number of states that would allow for segregation of state-action pairs dedicated to free (states 2,3,7) and forced (states 4,5) dLP and iLP choices. The same task state map was used to quantify the choice patterns on both the real and simulated sessions.

The RL simulations using this task state map were based on a Q-learning framework^32^. Each simulation run began with all Q-values set to 0 with the agent in state 1 (Figure 2) and ival set to 3. Because dLPs had a fixed payout, the goal of the agent was to maximize ival. The value of the ival parameter was determined as in the real task sessions in that it was decreased by each iLP and increased by each dLP.

Formally, the model had 3 main variables that governed choices and Q-value updating – ε-greedy, gamma (γ) and alpha (α). These parameters were set to predetermined values prior to each simulation run. The ε-greedy parameter governed the choice behavior of the agent while ε and α governed learning (i.e. Q-value updating). Specifically, ε-greedy determined the likelihood that the agent would choose the option with the highest Q-value (i.e. exploit) or randomly pick an option (i.e. explore). Prior to the choice, a random value between 0–1 was chosen (the *randi* command in MATLAB was used for all random draws and divided by 10). If this value was less than ε-greedy (or the Q-values of the 2 choice options were equal), the choice between the immediate (*iLP*) or the delay (*dLP*) option was determined randomly. Conversely, if the random value was greater than ε-greedy, the state-action pair with the highest Q-value was chosen and the agent performed the action and moved to that state.


Equation 1 $$\text{rand}[0\ldots 1]\{\begin{array}{l} \leq \varepsilon \to \text{rand}(\text{iLP},\text{dLP})\\ >\varepsilon \to \text{max}\left(Q_{im},Q_{de}\right) \end{array}$$

After every choice step, ival was updated according to the rules of the task described above, whereas Q-values were updated as described below. Once a choice was made, γ and α affected how the Q-values were updated. The first parameter, γ determined the impact of Q-values up to two trials in the future on Q-value updating. The second parameter, α, was the learning rate parameter and it determined the magnitude of the change in the current Q-value on each update. Specifically, once a choice was made (e.g. action) on some trial (*t*), the Q table was updated according to the value of the chosen next state and the possible states that lay beyond it, based on the following equation:

Equation 2 $$Q^{\text{new}}\left(cs_{t},a_{t}\right)=Q\left(cs_{t},a_{t}\right)+\alpha \cdot \left(\text{fival}\;+\gamma (\text{fqmax})-Q\left(cs_{t},a_{t}\right)\right)$$

Where *Q*(*cs*_*t*_, *a*_*t*_) was the Q-value of the current state (*cs*_*t*_) given some action (*a*_*t*_) that moved the agent to the next chosen state and resulted in an updated Q-value (*Q*^*new*^(*cs*_*t*_, *a*_*t*_)). *fival* and *fqmax* were determined by the best future state option; where *fqmax* was the future state option with the largest Q-value and *fival* was the ival that would be produced if the agent were to choose *fqmax*. The future state options were the Q-values that corresponded to state options that lied two trials in the future of a given choice. If both possible choice options had equal Q-values, *fqmax* was chosen randomly. Q-values were always scaled to a maximum of 6.

**Figure 1:**
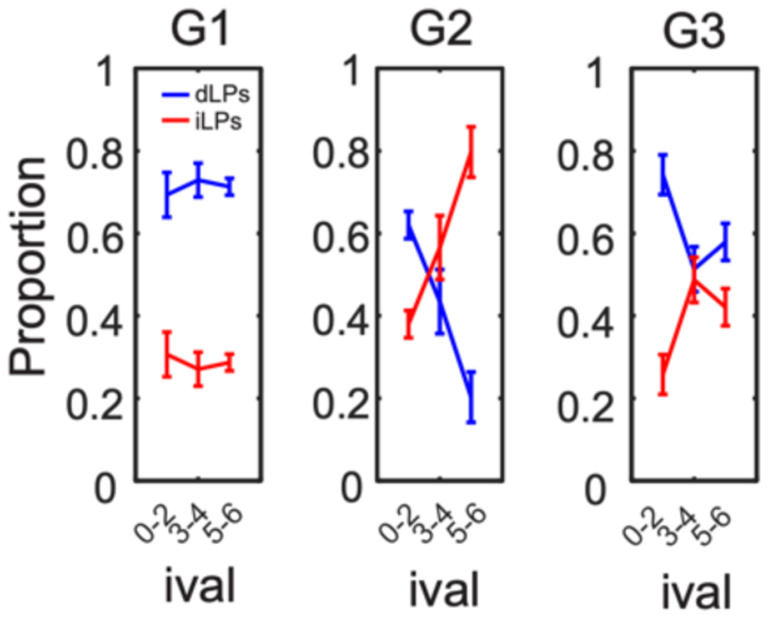
Different forms of cognitive effort as expressed in different behavioral strategies. Sessions were grouped based on the relative strength of a dLP-biased strategy versus an ival-tracking strategy in choice patterns. A strong bias for dLPs was observed in G1 that remained consistent regardless of ival. In contrast, G2 sessions exhibited a strong ival-tracking strategy as the ratio of dLPs:iLPs reversed when ival went from low to high. Finally, G3 exhibited a mix of both strategies such that there were more dLPs when ival was low and the ratio of dLPs to iLPs decreased at higher ivals but never exceeded 0.5.

The RL model was used to simulate the behavior of G1 and G2 sessions. G1 and G2 sessions were defined according to the behavioral strategy the rat’s employed which was in turn dictated by the length of the delay associated with the dLP option. There was a steady focus on dLPs in G1 sessions, whereas in G2 sessions, the focus on dLPs waned when ival was high (Figure 1). To capture this dynamic, a *dbias* term was added to the model such that whenever ival was >=3 on exploit trials, the Q-value associated with any dLP option was multiplied by 0.3–1, making it less likely that dLPs would be chosen. Importantly, *dbias* had no direct effect on how the Q-table was updated after a choice.

Each simulated session involved 20–30 runs of 50–58 trials to roughly match the average number of trials (choice+forced) in the actual sessions. The number of times each state was visited across all runs was tallied for both the real and simulated sessions. The state frequency visitation values were then normalized by dividing by the number of visitations of the most frequently visited state.

In order to evaluate how the parameter settings affected the choice behavior of the simulated agent, α and ε-greedy were independently varied between their minimum (0) and maximum (1) values in 0.1 increments. The parameter settings which resulted in choice preference ratios that most closely matched the real sessions were determined and the behavior of the model under these parameter settings were plotted.

### Ensemble tracking of ival

Two types of approaches were used to assess the tracking of ival by ensembles. The goal of the first approach was to determine the strength of ival tracking relative to all influences on activity throughout a session. This was done by applying Principal Components Analysis (PCA) to all time bins (200 ms/bin) throughout a session. The portions of the PCs associated with the epochs of interest were then extracted. The epochs of interest were the LP epoch (−1s to 0s prior to pressing the choice lever) and the outcome epoch (0 to 4s after the first pellet dropped). The second approach employed a supervised learning method in order to identify the ensemble patterns that most closely tracked with ival during the LP epoch. For each neuron, the activity in the 5×0.2s time bins preceding each LP was averaged. These values were concatenated into a single vector/neuron and these vectors were concatenated into a single matrix/session that was submitted to Maximally Collapsing Metric Learning (MCML; https://lvdmaaten.github.io/drtoolbox/)^33,34^ using ival on each trial as the class labels. MCML attempts to map all points in the same class into a single location in feature space while mapping all points in other classes to other locations. It does this by finding weights that minimize the Kullback–Leibler divergence in Mahalanobis distances between points within a class while maximizing the divergences between classes. Weightings are determined iteratively via gradient descent. Like PCA, it finds a set of components that in this case, varied across trials with ival but has been shown to be superior to PCA for this type of application^33^. The r^2^ between ival and the MCML component that most closely tracked ival in each session were compared across groups using a one-way ANOVA followed by post-hoc multiple comparisons.

### Single neuron tracking by ival

The mean spike count during the LP epoch (−1 to 0s) of all trials was z-scored and the ival vector denoting ival on each trial was normalized from −1 to 1. If the correlation between ival and the spike count was negative, the spike count vector was flipped to ensure all comparisons were made on equal ground. A linear regression model was used to fit the zscored spike count vector to the normalized ival vector (using *regstats* in Matlab). This generated a residual vector which denoted the quality of the fit on each trial. These residuals were stratified based on group, LP type and ival and were compared using a one-way ANOVA and post-hoc multiple comparisons with alpha=0.01. To be included in these analyses, the neuron had to have a mean spike count >0.1 across all LPs and a regression r^2^ value >0.2.

### Analysis of theta oscillations

Local field potentials (LFPs) were acquired in each of the electrophysiology animals. For analysis, the 64 LFPs were averaged, and analysis was performed on this signal from each recording. Signals were down sampled to 1000Hz and the time around each choice (−10 sec: 20 sec) was extracted. Spectral decomposition was performed via short-time Fourier transform over 0.5 s windows with 90% overlap. Real components of the signal were extracted and power in the theta band was taken as the average power for each time bin in the 6–12Hz band. Power measures were smoothed via moving average over 50ms for each trial.

### Analysis of oscillations in neural firing

Autocorrelations were computed for each neuron over +/−1s and binned at 1ms. PCA was then performed on autocorrelations (Figure 7), and it was found that PC3 split the neurons that exhibited 4–5Hz or theta oscillations. This was validated by examining the spectrum (via Fourier transform) of the mean autocorrelation obtained from autocorrelations with either positive or negative coefficients. An examination of the distribution of coefficients associated with PC3 yield a distribution with three clear modes. Positive loading neurons (>0.015) exhibited 4–5Hz oscillations and negative loading neurons (<−0.015) exhibited theta oscillations. In addition, an intermediate group with no clear oscillations was observed. Neurons that belong to each mode were then stratified by group as defined above (G1, G2, G3).

## Results

The goal of the study was to understand how the ACC represented different forms of cognitive effort. The adjusted-reward delay-discounting (DD) task could be solved using two main behavioral strategies that require the deployment of different forms of cognitive effort and that are associated with different behavioral choice patterns. The first strategy involves simply focusing on the delayed lever throughout the entire session. This strategy ‘dLP-biased’ strategy would produce the largest overall pellet yield but requires a resistance-based form of cognitive effort in order to wait out the delays associated with each dLP. If the waiting out the delays are too aversive, the alternative strategy would be to shift between levers based on their relative value, focusing on the immediate lever when ival is high and the delayed lever when ival is low. This strategy poses its own challenges as it requires a resource-based form of cognitive effort to continually keep track of value of the immediate lever. The behavioral manifestation of a dLP-biased strategy would be a high dLP:iLP on all free choice trials whereas an ival-tracking strategy would be manifest as a positive relationship between ival and the relative proportion of iLPs (i.e. ival/iLP slope).

The dLPs:iLP and the ival/iLP slope were calculated for each of the 54 sessions and the sessions were sorted using k-means clustering. The first cluster (G1), exhibited a clear dLP-biased strategy as there was a high dLP:iLP all every level of ival. This cluster contained only sessions with a 4 sec delay (Figure 1). The second cluster (G2), exhibited clear evidence of an ival-tracking strategy, as the relative proportion of iLPs depended strongly on ival. It contained only sessions with the 8 sec delay. The final cluster (G3) of sessions exhibited a mixture of the 2 strategies and was not limited to sessions of a specific delay length. Given the goal of understanding how ACC ensembles represented the two forms of cognitive effort mentioned above, the remainder of the study will focus exclusively on G1 and G2.

**Figure 2:**
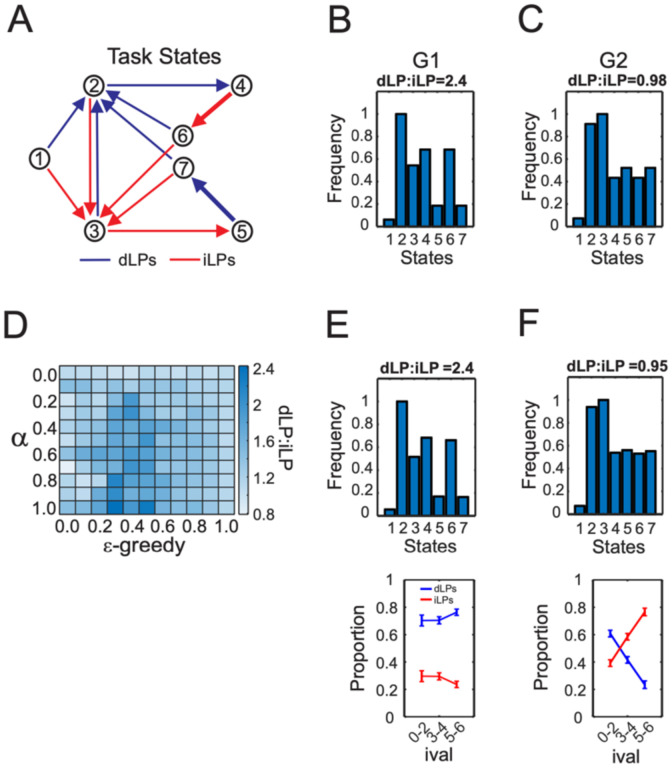
Quantifying choice behavior using a RL model. **A)** The transition matrix showing all possible task states. Task states were defined according to all the possible lever-press to lever-press choice transitions for the task. The sessions always began from an initial state (state 1), which would be equivalent to the pre-task period. The rat (or agent) could then choose to perform either a dLP (blue) or iLP (red) until they made repeated presses on either lever, which would then initiate a forced choice trial (thick lines with open arrows, states 5**→**7 or 4**→**6). Task state distributions of G1 **(B)**, G2 **(C)**. **D)** Parameter space of the effects of changing the learning rate (α) and the likelihood of exploration (ε-greedy) on dLP:iLP. The heat plot gives the free choice dLP:iLP produced by different model parameters. γ was held at 0.2 for all simulations. **E)** Simulations that matched the dLP:iLP of G1 (Left), G2 (Right) sessions were obtained using the same RL parameters (α=0.95; ε-greedy=0.4; γ=0.2) but a dbias term of 0.4 was included for the simulations shown in the right panel. In B and D, the task state frequency distributions were averaged across sessions and normalized to the maximum state visitation frequency.

Reinforcement Learning (RL) models have proven effective in providing theoretical accounts of ACC function related to cognitive control^26,35–39^. Therefore, a RL-based framework was used to gain further insights into the choice behavior on this task. Specifically, a Q-learning^32^ framework was employed where the agent transitioned from one state to the next state by performing an action (Figure 2A). Each state-action pair had a value (i.e. a ‘Q-value’) that was updated according to the current value of the immediate lever (i.e. ival). The task was divided into 7 state-action pairs as depicted in the task state map shown in Figure 2A, where the *actions* were the LPs and the *states* could be thought of as any time between pairs of LP actions. The states and the transitions between them captured every possible scenario permitted under the rules of the task (see Methods). A basic premise of the task map was that even numbered states represented free-choice dLPs whereas odd numbered states involved free-choice iLPs. The importance of either strategy was then evaluated by manipulating the specific model variables outlined below and comparing the choice behavior (i.e. the dLP:iLP ratio) of the agent with that of the animals in G1 and G2.

The dLP:iLP extracted from the G1and G2 are shown in Figure 2B,C. Since the G1 group exhibited a very high dLP:iLP, the state frequency distribution had far more even-numbered states than odd-numbered states. Furthermore, the higher frequency of state 3 relative to states 5 and 7 indicated that when the rats in G1 did make iLPs they tended to make one-off iLPs rather than consecutive ones. By contrast, the task-state distribution for G2 was characterized by a much higher prevalence of odd-numbered states and in particular states 5 and 7, which indicated more consecutive iLPs.

We then attempted to simulate the choice behavior of G1–G3 using an RL model based on the same state map shown in Figure 2A. The agent’s goal was to maximize ival by tracking/learning from outcomes and using this information to alter its choices. Its choice behavior was governed by the 3 parameters standard to all RL models, α, ε-greedy, and γ. The α parameter determines the learning rate, or the magnitude of the change in the current Q-value, given an outcome. The ε-greedy parameter determines the likelihood that the agent would choose the option with the highest Q-value (i.e. exploit) or randomly pick an option (i.e. explore) at the choice point of each trial. Finally, the γ variable determines how much future rewards were discounted when updating Q-values. We found that γ had relatively small effects on the behavior of the simulated agent, therefore it was held constant at 0.2 for all simulations described below.

After testing a range of model parameters (Figure 2D), a restricted range was identified that were capable of creating a dLP:iLP ratio and choice distribution similar to G1. This was obtained when α was close to 1 and ε-greedy was close to 0.4 (Figure 2D,E). In other words, when the agent exhibited robust learning from prior outcomes and made choices that balanced exploitation and exploration. In contrast, it was possible to attain a dLP:iLP similar to G2 using a much wider range of RL model parameters (Figure 2C), but all involved relatively low α and high ε-greedy values. However, none of these parameters (see Figure S1 for examples) allowed the model to capture the cross-over in choice distribution that characterized G2 (see Figure 1). We sought to understand why was this was the case.

There was no reason to believe that rats in G2 should have been less effective at learning from outcomes, especially considering that all animals had exposure to the 4s delay and many of the same rats contributed to both G1 and G2 sessions (Table 1). Rather, we conjectured that the critical difference between groups was that rats in G2 sessions actively avoided a dLP-biased strategy because they were unwilling to wait out the delays associated with the delayed lever. In other words, by increasing the delay from 4s to 8s, it effectively devalued dLPs. To capture this, a new parameter, *dbias,* was added to the model. The *dbias* parameter scaled down the Q-value of the dLP option whenever it was extracted from the Q-table on exploit trials. Importantly, it did not affect the actual values of dLPs in the Q-table nor did it impact how the Q-values were updated on each trial.

Using a uniform dbias to scale dLP independent of the current ival, it was still impossible to reproduce the choice behavior G2 (Figure S1). However, a uniform dbias does not accurately capture the choice behavior of G2, as dLPs were still the default option when ival was low (Figure 1). Therefore, we repeated the simulations but only applied the dbias term to dLP options that arose when ival was >3. In this case it was possible to accurately reproduce, the dLP:iLP, the state frequency distribution and the choice distribution of G2 (Figure 2F). In fact, this was possible using the identical RL parameters that were used to reproduce the choice behavior of G1 (Figure 2E). However, it should be noted that dbias had to be maximal in order to accurately reproduce G2 behavior, meaning that the value of dLP options arising when ival was 4–6, were scaled to 0. Collectively the results suggested that both groups were equally effective at using dLPs to maximize ival, but G2 deviated from this strategy by strongly avoiding dLPs when ival was high. This strategy struck a balance between maximizing rewards and minimizing delays.

**Figure 3:**
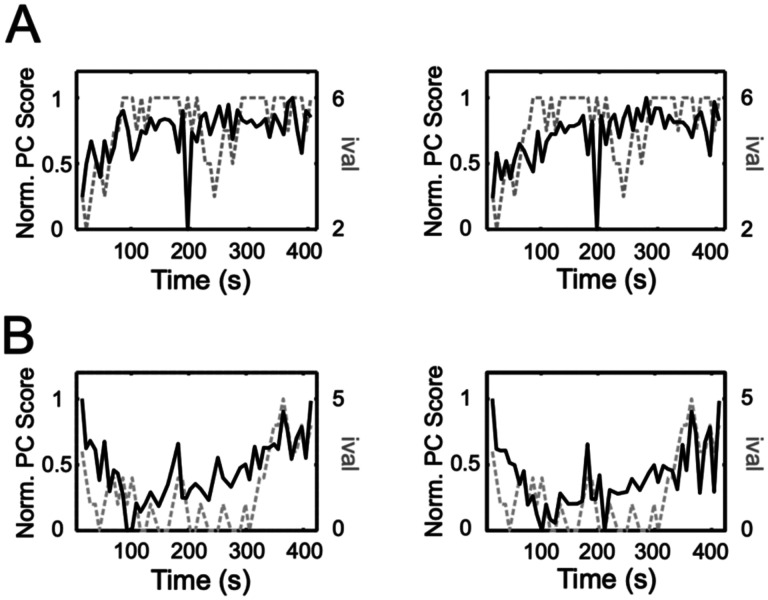
Neural representations of ival tracking. Examples of PCs (dark traces) that tracked ival (gray dashed traces) during various epochs of a session from **A)** G1 (PC1) and **B)** G2 (PC3). The left panels show the portion of the PC associated with the LP epoch and the right, with the outcome epoch. To help visualize the relationship between a given PC and ival, the PC was normalized between 0–1.

The RL simulations revealed that the strategies of both groups relied on ival in order to maximize rewards and, in the case of G2, to also minimizing waiting. We therefore searched for a neural representation of ival in the ACC recordings. The first approach involved applying Principal Component Analysis (PCA) to the spike count matrices that included all time bins (0.2s) throughout a given session. The portions of the PCs related to the LP epoch (−1 to 0s before the LP) and the outcome epoch (0 to 4s after the first pellet dropped) were then extracted and plotted alongside ival. The examples shown in Figure 3 highlight that PCs could be found in all 3 groups that tracked ival consistently across multiple task epochs.

**Figure 4:**
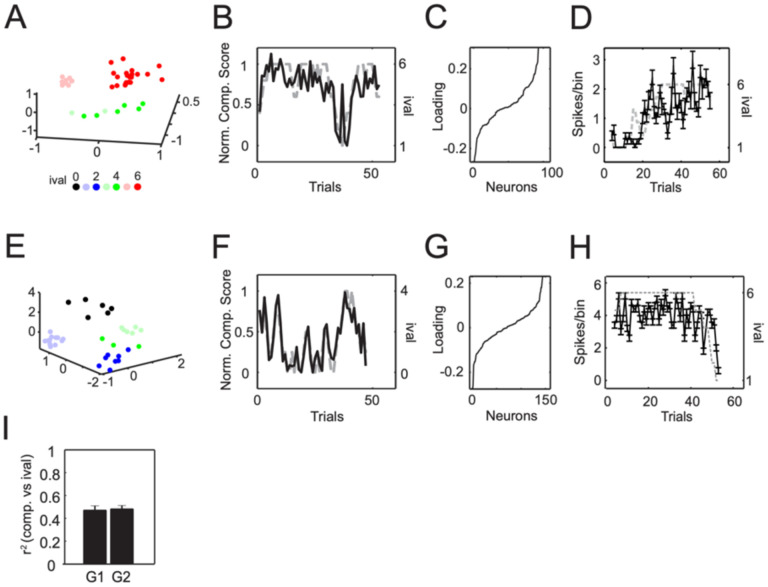
Robust ival tracking was present in all 3 groups. **A)** MCML was performed on concatenated spike count matrices of the LP epoch for an example session from G1. The clusters were colored according ival on the trial in which the LP was performed. **B)** The MCML components tracking ival across trials from another G1 session. **C)** The sorted loadings of neurons on the components shown in (B). **D)** Examples of neurons exhibiting ival tracking. The mean (and s.e.m.) spike rate/0.2s bin in the 1s period preceding the LP on each trial is plotted in black and ival is given by the gray dotted line. **(E-H)** same as (A-D) but for G2. **I)** The mean (and s.e.m.) r^2^ between ival and the ival-tracking component associated with the LP epoch for all sessions in G1 and G2.

Although Figure 3 provided evidence of ival-tracking, session-wide PCA was not optimal for identifying this signal since it is an unsupervised dimensionality reduction method and since the signal may not be equally strong in all time bins. A better approach would be to focus on the lever-press (i.e. choice) epoch and to use a supervised dimensionality reduction/classification method that optimizes projections of the ensembles to resolve information related to ival. Maximally Collapsing Metric Learning (MCML)^33,34^ was chosen for this purpose. First, the spike counts for each neuron during the LP epoch were averaged and then concatenated across trials to form a single vector/neuron. These vectors were then combined into a matrix and submitted to MCML, using ival as the class labels. The strength of ival tracking was quantified as the correlation between the MCML component and ival. In all groups, ensemble activity was found to distribute into separate clusters based on ival (Figure 4A,E). When the MCML components were plotted through time, the fidelity of ival tracking was striking (Figure 4B,F). The distribution of neuronal loadings on the ival-tracking components was approximately continuous, suggesting that ival-tracking was a property of the ensemble rather than a handful of specialized neurons (Figure 4C,G). Nevertheless, individual neurons varied in the strength of ival-tracking and examples of neurons loaded strongly onto the ival-tracking component are shown in Figure 4D,H. However, the strength of the correlation between ival and the MCML component did not differ by group (Figure 4I; F(1,30)=0.27,p=0.6). While this was somewhat surprising given the profound differences in the choice patterns of the two groups, it was accurately predicted by the results of the RL modeling above.

For the present purposes, ival-tracking could be considered a reasonable correlate of lever value. Although ival was tracked robustly, the fidelity of this tracking nevertheless fluctuated from one trial to the next. If we assume that the ival-tracking signal indeed provided an internal representation of lever value, then any deviation between this signal and ival would imply that the neuron had misrepresented lever value on that trial. By quantifying the fidelity of ival tracking, we could therefore estimate how each neuron represented lever value on each iLP trial. For these analyses, a linear regression model was used to fit the z-scored mean spike count during the LP epoch with ival. We then compared the residuals on low (0–2), medium (3–4) or high (5–6) ival trials in G1 versus G2 (in order to compare all neurons on equal grounds, the spike count vector was flipped if it was negatively correlated with ival). Using this approach, a positive residual on a given trial would imply that the neural representation of lever value was higher than what would be predicted from ival, whereas the opposite would be true of a negative residual.

**Figure 5:**
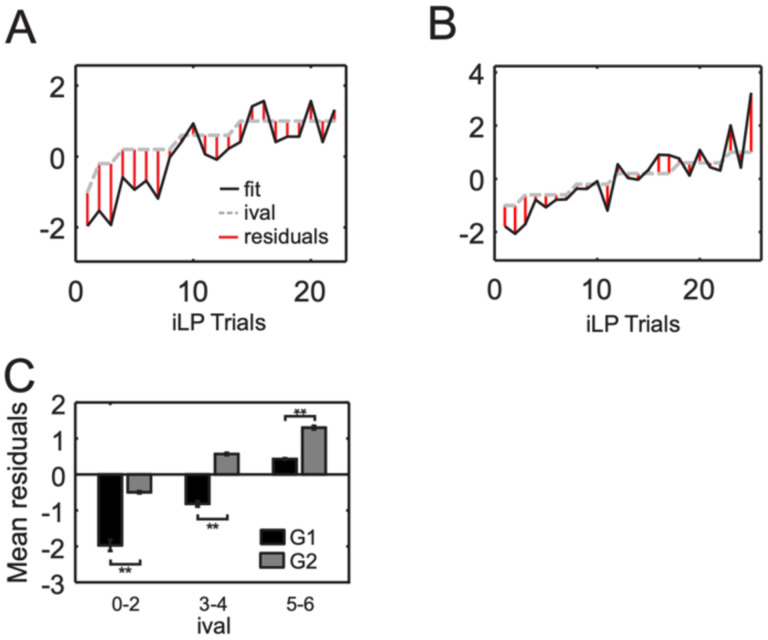
Group differences in tracking of ival by single neurons. For each neuron, a linear regression model was used to fit the z-scored mean spike count during the LP epoch across trials with ival. **A)** Example of a neuron from G1. **B)** Example of a neuron from G1. In (A) and (B), model fit line is black, the normalized ival line is dotted gray, the residuals on iLP trials are given by the red vertical lines. The sign of the residuals relates to whether the fit line was below ival (negative residual) or above ival (positive residual). **C**) Summary of the mean of the residuals on all iLP trials for G1 (black bars) and G2 (gray bars). Neurons had to have a mean spike count of at least 0.2 spikes/bin during the LP epoch of all trials and an r^2^ of 0.2 or greater to be included in (C). ** denotes p<0.001 based on post-hoc multiple comparison testing (Tukey’s HSD).

As shown by the two examples in Figure 5A–B, ACC neurons do a good job at tracking ival, but the residuals reveal that they are not perfect in this regard. Overall, there was a difference in the size of the residuals derived from model fits to neurons in G1 and G2 (F(5,2166)=335, p<0.0001). Post-hoc multiple comparisons showed that when ival was low (0–2), the residuals were significantly more negative in G1 than G2 (Figure 5C). Furthermore, the residuals remained negative as ival rose to 3–4 in G1, whereas they flipped to positive for G2. This implied that neurons in G1 uniformly under-valued iLPs across a wider range of trials than in neurons in G2. When ival was high (5–6), the residuals in both groups became positive, but they were on average significantly more positive in G2, suggesting that this group significantly over-valued iLPs when ival was high. Collectively, these results suggest that neurons from rats in G1 tended to under-represent the value of the immediate lever across a wide range of ivals, whereas neurons from rats in G2 systematically over-represented the value of the immediate lever as ival increased. Since the rats faced a choice between iLPs and dLPs on each trial, a uniform under-valuation of iLPs (as in G1) would presumably cause rats to always favor dLPs, whereas a relative over-valuation of iLPs at high ival (as in G2) would presumably have the opposite effect and create the cross-over profile shown in Figure 1.

**Figure 6:**
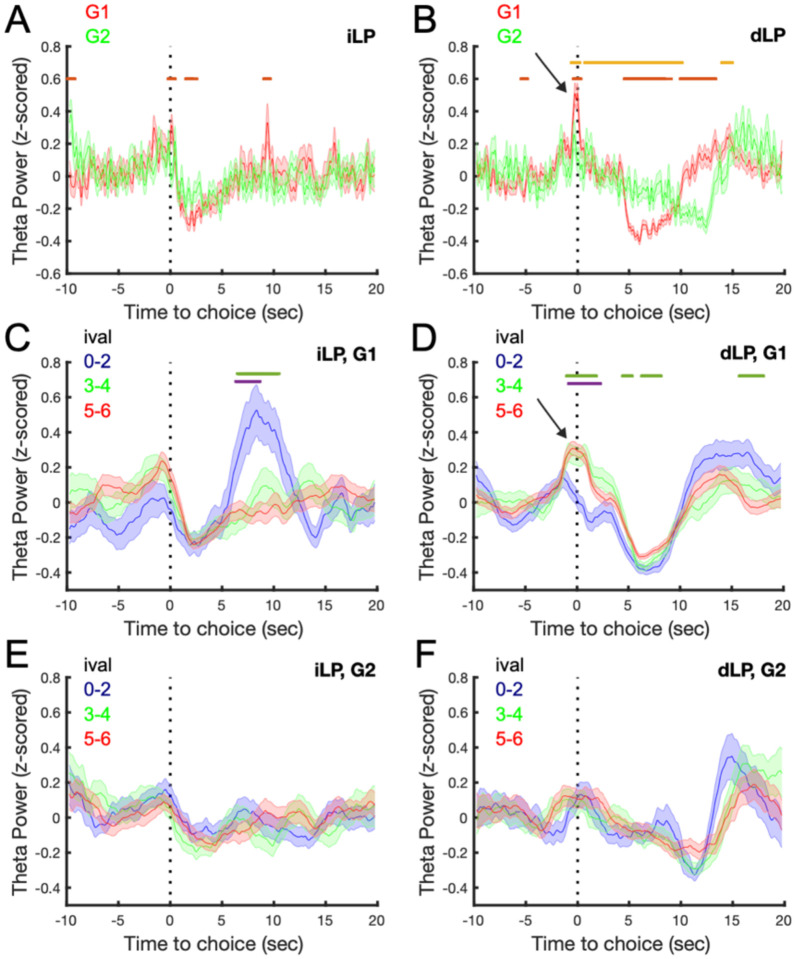
Theta power increases prior to delay choices when it is the preferred option. Theta oscillations were examined prior to iLPs (A) and dLPs (B). Theta power was highest in G1 prior to a dLP when compared with G2 or an iLP in G1. Stratifying theta power by ival revealed increases ~10 sec following an iLP in G1 for low ivals (C). Increases in theta power around a dLP were observed for mid and high ivals (D). While no effect of ival was observed for iLPs (E) or dLPs (F) in G2. Data are presented at mean ± SEM. Red line denotes Tukey’s HSD, p < 0.05 G1 vs G2; Orange line denotes Tukey’s HSD, p < 0.05 G1 dLPvs G2 dLP (A, B). Green line denotes Tukey’s HSD, p < 0.05, 0–2 vs 5–6 ival; purple line denotes Tukey’s HSD, p < 0.05, ival 0–2 vs 3–4.

In the human literature, there is a growing interest in the role of frontomedial theta in cognitive control^26–29^. These studies typically involve macroscopic measures of cortical activity, such as EEG. This motivated us to explore whether neural activity patterns reflecting a control signal could also be observed in the spectrum of the local field potentials (LFPs) and spike trains within the rat ACC. An assessment of the power spectrum in the LFP’s around the choice revealed several changes in theta that were most prevalent in G1 - the group hypothesized to use a resistance-based strategy. First, increases in theta power were observed in G1 immediately prior to a dLP (Figure 6B), but not an iLP (Figure 6A; group × choice × time interaction, F(11,12744)=5.09, p<0.0001). Theta power then declined immediately after an iLP (Figure 6A). However, following a dLP, theta power remained steady during the same period, only dropping after the termination of the 4 sec delay in G1 and the 8s delay in G2 (Figure 6B). Theta power then rebounded ~5 sec after the termination of the delay, which corresponds to the time during the intertrial interval. These data suggest that theta power may reflect a control signal associated with committing to wait for the delayed reward that turns off following reinforcement.

If theta power is important for facilitating a resistance-based control signal, then it should also be modulated by ival. Choosing a dLP when the ival is high is a difficult choice because a similar payout could be obtained with an iLP in the absence of a delay. Therefore, dLPs should require more resistance-based control when ival is high. Theta power was higher at medium and high ivals at the time of the lever press on dLP trials, when compared with low ival trials in G1 but not G2 (Figure 6C,D; ival group × choice × time interaction, F(22,12744)=2.3, p=0.0005). In G1, an increase in theta power was also observed but only following the iLP and only on low ival trials. Since choosing the immediate option at a low ival is a poor choice, this increase may reflect a completely separate negative feedback signal, potentially important for control more generally.

**Figure 7:**
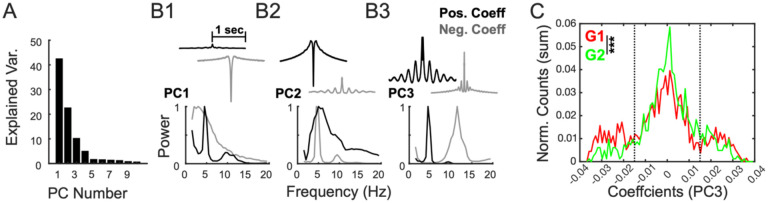
Increase theta entrainment of spiking is observed in G1. PCA was performed on the autocorrelations from the spike trains of each neuron and the amount of variance captured by each PC is shown (A). The first three PCs are shown in (B), the black and gray traces on top show the mean autocorrelation pattern captured by the positive and negative coefficients, respectively. The bottom panels show the spectrum of the mean autocorrelation for positive and negative loaders for each PC. Neurons that oscillate in the theta band were separated via negative coefficients on PC3 (B3). The distribution of coefficients for PC3 separated for G1 and G2. The three modes in the distribution made it possible to quantify the negative loaders on PC3 as theta entrained and compare between G1 and G2 (C). *** Kolmogorov-Smirnov test, p<0.0001.

To determine if changes in the theta oscillations were observable in spike trains, autocorrelations were examined for evidence of oscillatory entrainment in the theta band. PCA of the spiking autocorrelations from each neuron revealed two prominent oscillations, one in the 4–5Hz band and one in the theta band (Figures 7A, 7B). PC3 was found to separate neurons exhibiting theta band oscillations (Figures 7B3), which were identified by negative coefficients (<−0.015). An examination of the coefficients associated with PC3 yielded a distribution with three modes (Figure 7C). The distribution of coefficients for PC3 differed for G1 vs G2 (Two-sample Kolmogorov-Smirnov test, D=0.1354, p=1.27×10^−8^), where G1 was characterized by more negative coefficients and therefore more neurons exhibiting theta oscillations in spiking activity. These data indicate that theta oscillations in firing were more pronounced in G1 and therefore this candidate resistance-based signal is observable in both LFPs and spike trains.

## Discussion

In the present study, the adjusting amount delayed-discounting task was used to search for neural signals in ACC related to cognitive control. Sessions were divided 2 main groups, according to whether the primary strategy involved ival-tracking or a dLP-bias. By simulating these two strategies within an RL-model framework, it was possible to reproduce the choice patterns of both groups, indicating that these strategies provided a sufficient description of the behavior on this task. A neural signal related to ival-tracking was clearly observed in both groups. Evidence of a dLP-bias signal could also be found at the single neuron and LFP level but only for the group whose choice behavior was dominated by a dLP-biased strategy. These results provide insights into how ACC networks encode cognitive effort in the service of cognitive control.

One way to define cognitive effort is in terms of the domain that is being taxed (e.g. attention, memory, problem solving). However, it can also be defined in a more descriptive sense, emphasizing the process rather than the domain. In this framework, a *resourced-based* form of cognitive effort is utilized whenever a valuable but limited capacity resource, such as attention or working memory, is used to solve a task^1,3,4^. In contrast, a *resistance-based* form of cognitive effort is used in order to overcome some type of internal ‘resistive force’, such as unpleasantness or impatience^2,5^.

Typically, a resistance-based form is most relevant to delay-discounting tasks as effort is needed in order to wait out delays and resist the temptation of the immediate reward^40,41^. However, both types of cognitive effort are required for the adjusted amount delay-discounting task because this task incorporates the dynamic variable ival, which increases with every dLP and decreases with every iLP. A food-deprived rat seeks to maximize reward and avoid delays. By keeping track of ival, it would know when the payout of the immediate lever was high enough to warrant a switch from the delayed lever and therefore avoid the associated delays, without sacrificing pellet yield. However, ival-tracking strongly taps a resourced-based form of cognitive effort since it places a high demand on attention and mneumonic resources. Alternatively, a resistance-based form of cognitive effort is needed if rats opt for a dLP-biased strategy because this forces them to wait out all the delays associated with the delayed lever. Here we found that the dLP-biased strategy predominated when the delays were short (4s), whereas ival-tracking predominated when the delays were long (8s).

The modeling results suggested that a simulated RL agent could solve this task by simply tracking ival, although the agent’s choice behavior varied greatly depending on how strongly ival influenced choice. Consistent with model predictions, we found a strong neural signal within the ACC that closely tracked ival and this signal also influenced choice to varying degrees in different groups. This signal appeared to most strongly affect the choice behavior and the choice-related neural activity of G2. In particular, neurons derived from this group appeared to relatively undervalue iLPs when ival was low and relatively over-value iLPs when ival was high. An ival-tracking signal was also prominent in G1 animals, but neurons seemed to uniformly under-value iLPs regardless of ival. This is consistent with the RL modeling as a novel dLP bias term was needed to accurately simulate the behavior pattern of G1. These signals map onto the two forms of cognitive effort outlined above: The strong dLP bias of G1 would require the deployment of a resistance-based form of cognitive effort to overcome the delay-to-reward associated with each dLP, whereas G2 would need to rely more heavily on the deployment of the resourced-based form of cognitive control in order to ensure that ival predominately influenced choice.

The present results are therefore consistent with theories highlighting a role of the ACC in cognitive control^1,10,42,43^. Cognitive control processes, mediated by the ACC, are thought to dynamically regulate how much and what type of cognitive effort should be deployed based on the expected value of the outcome, relative to the cost of implementing and maintaining the cognitive effort. Indirect support for this theory comes from human imaging studies^44^ and electrophysiology studies in primates^45^ and rodents^46^ showing that ACC neurons encode the value of outcomes. ACC neurons potentially compute value by combining information about the magnitude of a reward^47^ and the spatial or temporal distance to it^48,49^.

There is also clear evidence for a role of the ACC in signaling effort. Lesions of the ACC make it less likely that rodents will choose an option that requires greater effort, even if that effort yields a higher reward^20–22,50^. In addition, neurons within the rodent and primate ACC signal the degree of physical effort, regardless of whether effort is defined in terms of the size of an obstacle, the angle of a ramp to be traversed, or in terms of competitive effort^17,45,51–53^. Most of these studies have found that ACC neurons encoded a multiplexed representation that combined information about relative effort and the relative value of the reward. Hillman & Bilkey^54^ referred to this multiplexed signal as representation of the overall net utility, which fits well with the description of value in the theories of cognitive control mentioned above.

In the only study addressing the role of the rodent ACC in cognitive effort, ACC inactivation was found to reduce the willingness of rats to expend cognitive effort as defined in terms of visuospatial attention on a variant of the 5-choice serial reaction time task^23^. Although no prior study to our knowledge has explored the cellular correlates of cognitive effort, Holroyd and colleagues^25,28^ investigated potential electrophysiological correlates in human EEG signals. They built on findings suggesting that the ACC is a main generator of ‘frontal midline theta’ and argued that this signal is related to the application of both physical and cognitive effort. They postulated that when this signal is combined with a second reward-related signal (also believed to be generated within the ACC), it creates a representation of expected value or net utility. Their theory is supported by fMRI studies that have found that representations of anticipated effort and prospective reward elicit overlapping patterns of activation in ACC^19,55^.

We found that variations in theta exhibited characteristics that would be expected of a resistance-based signal. Specifically, the fact that theta power was most robust in G1 and only observed prior to delay choices fit the bill for a resistance-based control signal. In addition, theta entrainment of neural activity was lowest in animals that used a resource-based control strategy (i.e. G2). Collectively these data suggest that theta may provide a mechanism to implement a resistance-based control strategy.

Cognitive control theories are by definition, cognitive in nature. However, it difficult to know whether ACC neurons compute value or cost in a cognitive/economic sense or whether the ACC simply tracks the internal reactions to the results of such computations performed elsewhere in the brain. On one hand, the ACC is part of a cognitive control network that includes “cognitive” regions such as the posterior parietal cortex and prefrontal cortex^56^. On the other hand, it has extensive, often bi-directional connections, with subcortical, brain stem and spinal cord regions involved in tracking and modulating emotional state and autonomic tone^14–16^. Accordingly, the main effects of ACC stimulation are changes in autonomic markers such as heart rate, blood pressure and breathing^16,57^. The integration of ACC signals in this array of brain regions would likely require a mechanism to synchronize neural activity amongst them and theta oscillations would be a good candidate. This may facilitate the integration of cognitive, emotional, and autonomic signal across brain-wide circuits.

Prior work has implicated theta band synchrony in PFC with either the amygdala or the ventral hippocampus during periods of stress and/or anxiety^58,59^. Further, a reduction in synchrony in theta between these regions corresponds to a loss of control over stress and anxiety^58,60,61^. While it is difficult to equate 4 or 8 sec delays with an anxiety or stress producing stimulus, it is clear that the animals do not like it as they will avoid waiting for the delay when possible. Our data suggest that theta synchrony may be mechanism to mitigate the unpleasantness of the delay and therefore a good candidate of a resistance-based control signal.

One problem in determining whether recorded signals are cognitive or affective in nature, is that laboratory tasks, including the adjusted-reward delay-discounting task, use biologically relevant events as both a source of information and motivation. For instance, consider the observation that ACC ensembles robustly tracked ival across multiple epochs. By tracking this variable, rats could in theory create a relative value representation of both levers, informing them about when the expected payout of the immediate lever was high and therefore when it was advantageous to switch to this lever. However, ival was also a proxy of the recent reward history. Therefore, the ival-tracking signal could simply reflect the emotional or autonomic response to the ongoing tally of relative wins and losses. In terms of this question, it is worth noting that the ival-tracking signal appeared to only guide lever choices in G2 but not G1 (Figure 1), even though it was equally robust in all groups. This suggests that the ACC may always track expected value signals, regardless of whether they are used to guide decision-making or not.

Cognitive effort is costly because it is inherently aversive^4^. It has been argued that the cost of cognitive effort is ‘felt’ at a physiological level through changes in emotion and autonomic tone^2,4,12,13^. Therefore, ACC may assess the cost of cognitive effort in the same way it calculates the value of an expected outcome, which is indirectly via changes in autonomic tone. We would argue that this is generally the case, in that the primarily function of the ACC is likely to monitor and regulate autonomic tone^16^, but when these signals are transmitted to downstream regions, perhaps via theta oscillations, they serve as important cues that guide cognitive control and decision-making.

## Supplementary Material

1

## Figures and Tables

**Table 1:** Distribution of sessions/rat

Rat #	2	5	8	13	17	42	49	58	60
**G1**	0	2	0	1	4	4	1	4	3
**G2**	0	0	0	0	0	0	4	4	2
**G3**	1	0	1	0	1	4	5	2	6
