# Supplementary material for: Neural basis of cognitive control signals in anterior cingulate cortex during delay discounting
Source: bioRxiv. 2024 Jun 8:2024.06.07.597894. Preprint. [Version 1] doi: 10.1101/2024.06.07.597894 (PMC11185781; doi:10.1101/2024.06.07.597894)
Supplement: 1 [file NIHPP2024.06.07.597894V1-supplement-1.pdf]

bioRxiv preprint doi: https://doi.org/10.1101/2024.06.07.597894; this version posted June 8, 2024. The copyright holder for this preprint (which was not certified by peer review) is the author/funder, who has granted bioRxiv a license to display the preprint in perpetuity. It is made available under aCC-BY 4.0 International license.

Supplemental Figure

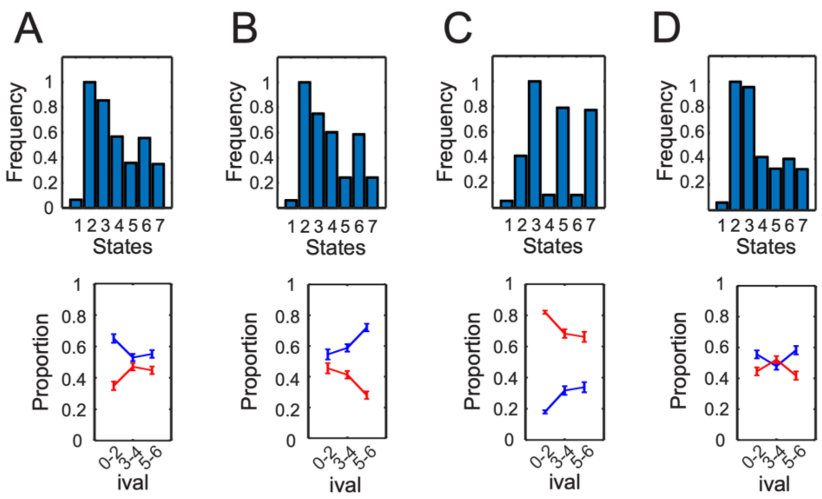

Figure S1: *Modeling approaches for G2. A) α=0.2, ε-greedy=0.8, no dbias. B) α=0.5, ε-greedy=0.5, no dbias. C) α=0.97, ε-greedy=0.4, uniform dbias=1. D) α=0.2, ε-greedy=0.8, uniform dbias=0.5. In each subpanel, the state frequency distribution (top) and choice distribution with respect to ival (bottom) are plotted as in Figure 2.*
